# Psychometric Properties of the Chinese Version of the Functional Assessment of Self-Mutilation (FASM) in Chinese Clinical Adolescents

**DOI:** 10.3389/fpsyt.2021.755857

**Published:** 2022-01-26

**Authors:** Diyang Qu, Yanni Wang, Zhiguo Zhang, Linlin Meng, Feng Zhu, Tiansheng Zheng, Kongliang He, Yue Zhou, Chuanxiao Li, He Bu, Yongjie Zhou

**Affiliations:** ^1^Shenzhen Mental Health Center, Shenzhen Kangning Hospital, Shenzhen, China; ^2^Vanke School of Public Health, Tsinghua University, Hong Kong, China; ^3^Department of Maternal, Child and Adolescent Health, School of Public Health, Lanzhou University, Lanzhou, China; ^4^Health Science Center, School of Biomedical Engineering, Shenzhen University, Shenzhen, China; ^5^Linyi Mental Health Center, Linxi, China; ^6^Suzhou Guangji Hospital, Suzhou, China; ^7^Wenzhou Kangning Hospital, Wenzhou, China; ^8^Hefei Fourth People's Hospital, Hefei, China; ^9^CAS Key Laboratory of Mental Health, Institute of Psychology, Chinese Academy of Sciences, Beijing, China; ^10^City University of Hong Kong, Hong Kong, China

**Keywords:** Functional Assessment of Self-Mutilation, validity, reliability, factor analysis, adolescent, Chinese

## Abstract

**Background:**

Functional Assessment of Self-Mutilation (FASM) is one of the most widely used tools assessing adolescent's non-suicidal self-injury. However, the Chinese version of FASM (C-FASM) is lacking. The present study aimed to adapt the FASM to the Chinese patients and examine its reliability and validity.

**Methods:**

The original English version of the FASM was translated into Chinese following Brislin's model of cross-culture translation, and then, pilot study and cognitive interview were carried out with 15 adolescent patients to assess the acceptability and comprehensibility of all items. The items were subsequently tested in a sample of 621 Chinese adolescent patients recruited by 20 psychiatric or general hospitals in nine provinces across China. We examined the distribution of responses for each item. Factor analysis, Cronbach's α and McDonald's Ω, intraclass coefficient, and Spearman's rank correlations were deployed to assess the dimensional structure, internal consistency reliability, test–retest reliability, and criterion validity.

**Results:**

The final adapted C-FASM included a 10-item method checklist and a 15-item function checklist of NSSI, and other characteristics of NSSI. C-FASM exhibited acceptable internal consistency (α = 0.81 and Ω = 0.80 for method checklist; α = 0.80 and Ω = 0.76 for function checklist) and test–retest reliability (method checklist: 0.79; function checklist: 0.87). Factor analysis for NSSI functions yielded a three-factor model with a good model fit. In addition, the instrument showed an expected correlation with the instrument of the Deliberate Self-Harm Behavior Inventory (*r* = 0.84, *p* < 0.001).

**Conclusions:**

The C-FASM has good content, structural validity, and reliability. The instrument can be helpful to Chinese adolescents as a comprehensive measure of NSSI behaviors.

## Introduction

Non-suicidal self-injury (NSSI) refers to the direct, deliberate self-harm of one's body tissue without suicidal intent, and the purposes are not socially sanctioned (1). NSSI behaviors are prevalent in adolescents, with a global 12-month prevalence of 14.2% (2), and appear to be increasing (3). NSSI behaviors in adolescents have fueled concerns in China due to the high prevalence, ranging from 12 to 24% in adolescents (4, 5).

Most assessments, such as the inventory of Statements of Self-Injury (6), Non-Suicidal Self-Injury Assessment Tool, and Deliberate Self-Harm Behavior Inventory (7), only assess the methods of NSSI. The Functional Assessment of Self-Mutilation (FASM) (8) measures the methods and functions of NSSI at the same time, providing essential measurement to guide the self-injury intervention (9).

FASM was designed by Lloyd et al. (8) and is widely used in assessing adolescent self-injury (9). The original FASM evaluates both the 11 types of NSSI behaviors and 22 function domains of NSSI (i.e., reasons for engaging in NSSI) that directly affect treatment (10–12). The short version of FASM is preferable to facilitate the inclusion of NSSI in a clinical setting (13).

The 22 NSSI function items in FASM were categorized into a four-factor model according to Nock and Prinstein (14): (1) automatic positive reinforcement (i.e., increase their positive feelings); (2) automatic negative reinforcement (i.e., a decrease of aversive feelings); (3) social positive reinforcement (i.e., increase of social events or attention from others); and (4) social negative reinforcement (i.e., decrease of undesirable social events). The model is the most widely used functional model of NSSI and has been confirmed among high school students in the United States (15).

However, the original FASM and function structures may be compromised by various issues, such as cross-cultural variations in the factor structure of function, and ecological validity of translated versions (9). For example, the four-factor model has been questioned in later studies, including “close to acceptable fit” issues (10) and failing to distinguish positive from negative reinforcement of interpersonal functions among Swedish adolescents (16). Above all, cultural differences have a considerable influence on the nature of NSSI. It is crucial to examine the reliability and validity of a translated tool before it is adopted in different culture. Yet, only a few studies have addressed the replicability of the FASM scale in Chinese cultural settings. Leong et al. noted that Nock and Prinstein's four-factor model did not reach adequate fit among Macao adolescents (17). You et al. (18) identified a three-factor model of NSSI functions using FASM among Hong Kong high school students. To the best of our knowledge, there is no tool research of the Chinese revision of FASM.

Meanwhile, NSSI might also vary over time since the FASM was developed two decades ago. Thus, a timely and modified version of FASM for Chinese population is required. In this study, we tested the FASM in the Chinese cultural background to assess the validity and reliability of the Chinese version of FASM (C-FASM) and examined the factor structure of NSSI function in a Chinese adolescent clinical sample.

## Methods

### Translation and Cross-Cultural Adaptation

The original English version of FASM was translated into Chinese based on the Brislin's translation model. Nine experts with knowledge of the English language translated this scale with the standardized forward-and-backward translation procedure to retain the scale's conceptual equivalent. After the translation, one methodologist, three psychologists, and two professional psychiatrists consolidated all the versions, discussed the cultural equivalence, and developed a satisfactory version for field testing (19). The cognitive interviews were carried out with outpatient and inpatient adolescents (*N* = 15) to ensure that patients could understand the items more accurately.

### Test Population

A cross-sectional survey was conducted to evaluate the psychometric properties of C-FASM.

The C-FASM items were tested in adolescents with a mental disorder who engaged in self-injury over the past 12 months. Participants were enrolled from 20 psychiatric or general hospitals in nine provinces across China to consider the difference in geography, economic development, and custom. Participants were recruited from August 2020 to November 2020. Inclusion criteria were as follows: (1) age 12 to 18 years, (2) was diagnosed with a mental disorder by senior psychiatrists according to the Diagnostic criteria and Statistical Manual of Mental Disorders Fifth Edition (DSM-5), and (3) had at least five times of non-suicidal self-harm behaviors in the past 12 months. Exclusion criterion was a history of intellectual disability. As a result, 621 adolescents were recruited in our study (93 boys and 528 girls), with an average age of 15.0 (SD = 1.7, range = 12–18, Supplementary Table 1).

The protocol of this study was approved by the ethics committee of the Institutional Review Board (IRB) of the Shenzhen Kangning Hospital (IRB: 2020-K021-01). Written informed consent was obtained from participants and their legal guardians.

### Instruments for Criterion Validity

#### Deliberate Self-Harm Behavior Inventory (DSHI)

Participant's methods and frequency of NSSI in the past 12 months were also assessed by the 17-item Deliberate Self-Harm Behavior Inventory (7). A higher total score of the DSHI indicates that an individual has engaged in a greater variety of NSSI with higher frequency. This scale demonstrated good psychometric properties among Chinese children (20, 21). In the present study, the Cronbach's α was 0.80, showing acceptable internal consistency.

### Statistical Analysis

The total sample was randomly split into two independent subsamples (sample 1 and sample 2) for the test–retest subsample to analyze the factor structure of NSSI function. Descriptive statistics were performed to examine the socio-demographic characteristics of sample 1 and sample 2. The distribution of responses, mean (M) and standard deviation (SD) values, skewness, and kurtosis were calculated for each of the FASM items for the entire sample. Factor analysis, internal consistency reliability, test–retest reliability, and criterion validity of C-FASM were evaluated in this study.

#### Factor Analysis

The Kaiser–Meyer–Olkin (KMO) value (≥0.5) (22) and Bartlett's test (<0.05) (23) were computed to evaluate the suitability of the data for factor analysis of FASM functions. Factor models for NSSI function were derived using Exploratory Factor Analysis (EFA) in the first randomly chosen subset (sample 1). Confirmatory Factor Analysis (CFA) and Exploratory Structural Equation Modeling (ESEM) were subsequently carried out on the second subset sample 2) to determine the replicability of the EFA results. An independent calculation of the partial item test was made to estimate the item discrimination rate in each dimension (24).

The Principal Axis Factor analysis with oblimin rotation (25) and varimax rotation methods were adopted for EFA. The number of factors was determined by eigenvalues and scree plots using an eigenvalue criterion higher than 1 (26). Items were removed when cross-load on more than one factor (>0.32) or <0.15 difference from an item's highest factor loading (27, 28).

In the CFA and ESEM analysis, model fit was evaluated using multiple indexes of fit, including elative/normed chi-square (χ2/df), comparative fit index (CFI), goodness-of-fit index (GFI), adjusted GFI (AGFI), Tucker–Lewis index (TLI), the standardized root mean square residual (SRMR), root mean square error of approximation (RMSEA), and Akaike information criterion (AIC). The criteria for well-fitting models were as follows: CFI ≥ 0.95, GFI ≥ 0.90, AGFI ≥ 0.85, TLI ≥ 0.95, SRMR <0.08, and RMSEA <0.06 (29). The items with lower factor loading (<0.32) would be removed.

#### Reliability and Criterion Validity Analysis

The internal consistency and test–retest reliability determined the reliability of the C-FASM. Internal consistency of FASM was assessed using Cronbach's α and McDonald's Ω coefficients (30). Alpha coefficient and McDonald's Ω higher than 0.70 indicated a reasonable internal consistency (31). Test–retest reliability was estimated by intraclass coefficient (ICC), calculated by the correlation between the first and second completion of the scale and adequate value above 0.60. The criterion validity was assessed via association between FASM and DSHI using Spearman's rank correlations. According to previous studies, we hypothesized that the FASM score should be positively related to the DSHI score.

All statistical analyses were performed using IBM SPSS version 20.0 software (SPSS Inc., Chicago, Illinois, USA) and AMOS (version 18.0, Smallwaters Corporation, Chicago, IL, USA).

## Results

### Content Validity in the Context of Chinese Culture

To ensure the validity of the C-FASM's content, a pilot study and cognitive interviews were conducted among 15 adolescents to test the content validity of C-FASM. Results from the pre-test were discussed by six experts in research, and some modifications were made accordingly. As recommended by experts, a common NSSI method (i.e., punched walls or objects) (32) among Chinese adolescents was added to the method checklist. However, only a few adolescents had engaged in “Burned your skin” (9.4%) and “erased your skin” (20.0%). A few adolescents had reported that they engaged in self-injury for “To be like someone you respect” (6.4%), “To feel more a part of a group” (17.4%), “To give yourself something to do with others” (12.9%), and “To make others angry” (9.7%). Thereby, the above items were removed due to the fewer reports by Chinese adolescents (not typical in Chinese culture) and high skewness and kurtosis (Tables 1, 2).

**Table 1:** Distribution of responses for FASM method checklist (*N* = 621).

**Method of NSSI**	**Frequency**, ***N*** **(%)**					**M**	**SD**	**S_**kewness**_**	**K_**urtosis**_**
	**0**	**1**	**2–5**	**6–10**	**≥11**				
1. Cut or carved on your skin	52 (8.3)	25 (4.1)	190 (30.6)	129 (20.8)	225 (36.2)	2.72	1.23	−0.683	−0.345
2. Hit yourself on purpose	228 (36.7)	15 (2.4)	164 (26.5)	81 (13.0)	133 (21.4)	1.80	1.56	0.093	−1.465
3. Pulled your hair out	326 (52.5)	15 (2.4)	132 (21.2)	50 (8.1)	98 (15.8)	1.32	1.54	0.619	−1.157
4. Gave yourself a tattoo	393 (63.3)	51 (8.3)	104 (16.7)	35 (5.6)	38 (6.1)	0.83	1.25	1.264	0.366
5. Picked at a wound	303 (48.8)	41 (6.6)	138 (22.2)	51 (8.2)	88 (14.2)	1.32	1.49	0.622	−1.06
6. Burned your skin	563 (90.6)	18 (2.8)	28 (4.6)	6 (1.0)	6 (1.0)	0.19	0.65	3.846	15.33
7. Inserted objects under your nails or skin	422 (68.0)	31 (5.0)	92 (14.8)	27 (4.3)	49 (7.9)	0.79	1.29	1.393	0.595
8. Bit yourself	258 (41.5)	33 (5.3)	159 (25.7)	67 (10.8)	104 (16.7)	1.56	1.52	0.345	−1.319
9. Picked areas of body	356 (57.3)	32 (5.2)	127 (20.5)	43 (6.9)	63 (10.1)	1.07	1.40	0.907	−0.579
10. Scraped your skin	270 (43.4)	32 (5.2)	136 (21.9)	73 (11.8)	110 (17.7)	1.55	1.56	0.361	−1.386
11. ‘erased’ your skin	496 (80.0)	19 (3.1)	54 (8.6)	27 (4.3)	25 (4.0)	0.50	1.08	2.079	3.101
12. Punched walls or objects	191 (30.8)	50 (8.1)	183 (29.5)	79 (12.7)	118 (18.9)	1.81	1.47	0.106	−1.299

**Table 2:** Distribution of responses for FASM function checklist (*N* = 621).

**Reason, *N* (%)**	**Never**	**Rarely**	**Some**	**Often**	**M**	**SD**	**S_**kewness**_**	**K_**urtosis**_**
1. To avoid school, work, or other activities	376 (60.5)	126 (20.2)	69 (11.2)	50 (8.1)	0.67	0.96	1.25	0.34
2. To relieve feeling numb or empty	144 (23.2)	124 (20.0)	171 (27.5)	182 (29.3)	1.63	1.13	−0.20	−1.36
3. To get attention	406 (65.4)	121 (19.5)	51 (8.2)	43 (6.9)	0.57	0.91	1.52	1.20
4. To feel something, even if it was pain	145 (23.3)	129 (20.8)	186 (30.0)	161 (25.9)	1.58	1.11	−0.18	−1.31
5. To avoid doing something unpleasant you don't want to do	305 (49.1)	129 (20.8)	104 (16.7)	83 (13.4)	0.94	1.09	0.73	−0.89
6. To get control of a situation	403 (64.9)	104 (16.7)	78 (12.6)	36 (5.8)	0.59	0.92	1.35	0.59
7. To try to get a reaction from someone, even if it's negative	399 (64.3)	124 (20.0)	63 (10.1)	35 (5.6)	0.57	0.89	1.44	1.00
8. To receive more attention from your parents or friends	380 (61.2)	124 (20.0)	69 (11.1)	48 (7.7)	0.65	0.96	1.27	0.40
9. To avoid being with people	335 (53.9)	113 (18.2)	105 (16.9)	68 (11.0)	0.85	1.06	0.86	−0.68
10. To punish yourself	161 (25.9)	139 (22.4)	145 (23.4)	176 (28.3)	1.54	1.16	−0.05	−1.44
11. To get other people to act differently or change	413 (66.5)	111 (17.9)	59 (9.5)	38 (6.1)	0.55	0.90	1.51	1.17
12. To be like someone you respect	581 (93.6)	25 (4.0)	8 (1.3)	7 (1.1)	0.10	0.43	5.01	26.76
13. To avoid punishment or paying the consequences	460 (74.1)	86 (13.8)	51 (8.2)	24 (3.9)	0.42	0.80	1.89	2.58
14. To stop bad feelings	75 (12.1)	106 (17.1)	187 (30.1)	253 (40.7)	2.00	1.03	−0.66	−0.78
15. To let others know how desperate you were	323 (52.0)	147 (23.7)	88 (14.2)	63 (10.1)	0.82	1.02	0.94	−0.40
16. To feel more a part of a group	513 (82.6)	63 (10.1)	32 (5.2)	13 (2.1)	0.27	0.65	2.61	6.30
17. To get your parents to understand or notice you	366 (58.9)	116 (18.7)	85 (13.7)	54 (8.7)	0.72	1.00	1.11	−0.10
18. To give yourself something to do when alone	354 (57.0)	129 (20.8)	78 (12.5)	60 (9.7)	0.75	1.01	1.08	−0.13
19. To give yourself something to do with others	541 (87.1)	51 (8.2)	20 (3.2)	9 (1.5)	0.19	0.55	3.27	10.89
20. To get help	444 (71.5)	96 (15.5)	53 (8.5)	28 (4.5)	0.46	0.83	1.76	2.07
21. To make others angry	561 (90.3)	42 (6.8)	14 (2.3)	4 (0.6)	0.13	0.45	3.87	16.18
22. To feel relaxed	153 (24.6)	116 (18.7)	164 (26.4)	188 (30.3)	1.62	1.16	−0.19	−1.41

Therefore, the initial version of the C-FASM comprised the 10 items method checklist and 18 items of NSSI function and other characteristics of NSSI.

### Factor Structure of NSSI Function

There were no significant differences in socio-demographic characteristics between sample 1 (*N* = 319, 16.3% boys) and sample 2 (*N* = 302, 13.6% boys, Supplementary Table 1).

#### Exploratory Factor Analysis

EFA was performed using the 18 items with scores of each item obtained from participants in sample 1. An EFA with the 18 items presented a four-factor model solution with eigenvalues > 1 (oblimin rotation: KMO = 0.84, χ2 = 1803.81, *p* < 0.001; varimax rotation: KMO = 0.83, χ2 = 1325.40, *p* < 0.001). Items with low factor loadings or substantial cross-loadings were removed sequentially. Therefore, item 18 (“To give yourself something to do when alone”) was removed due to the low factor loading (<0.32). Item 6 (“To get control of a situation”) and item 11 (“To get other people to act differently or change”) were removed due to the cross-loadings.

**Figure 1:**
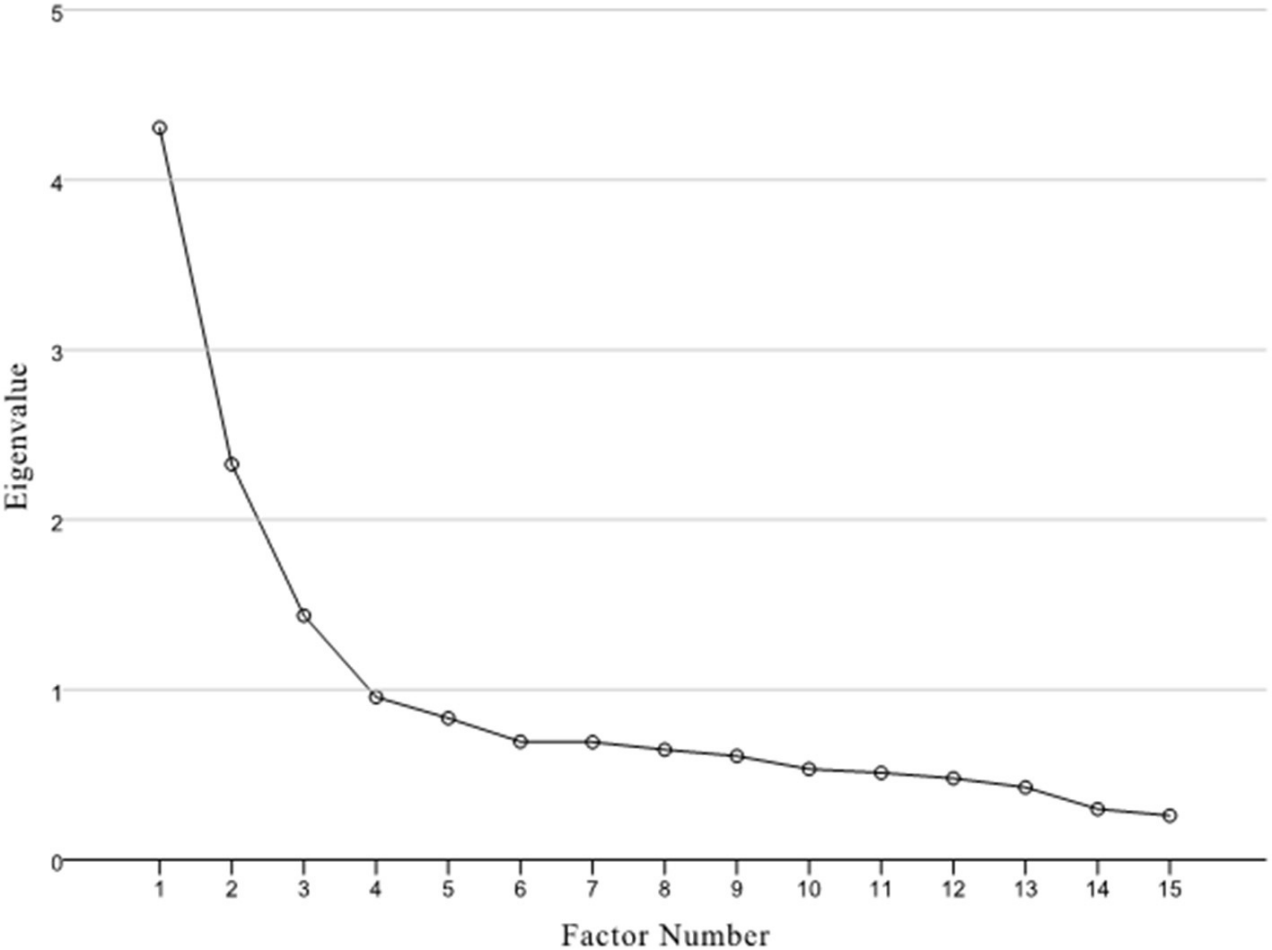
Scree plot for the EFA in Sample 1.

A second EFA without items 6, 11, and 18 was performed. As shown in the scree plot, the three-factor model with 15 items was better fit these results (KMO = 0.84, χ2 = 1640.91, *p* < 0.001, eigenvalues = 1.46, Figure 1). A satisfying three-factor solution was extracted with eigenvalues of 4.317, 2.327, and 1.493, which could explain 29, 16, and 10% of the observed variance. All factor loadings were higher than 0.50 (Table 3). Items 2, 4, 10, 14, and 22 were exclusive on factor 1; items 3, 7, 8, 15, 17, and 20 exclusively on factor 2; and items 1, 5, 9, and 13 were exclusive on factor 3.

**Table 3:** Standardized factor loadings for the three-factor models derived by EFA (*N* = 319) and confirmed by CFA and ESEM (*N* = 302).

	**EFA**			**ID**	**CFA**	**ESEM**
**Items**	**Factor 1**	**Factor 2**	**Factor 3**			
2. To relieve feeling numb or empty	0.74			0.55	0.70	0.67
4. To feel something, even if it was pain	0.60			0.16	0.63	0.71
10. To punish yourself	0.60			0.38	0.47	0.45
14. To stop bad feelings	0.71			0.45	0.47	0.52
22. To feel relaxed	0.77			0.50	0.55	0.56
3. To get attention		0.80		0.66	0.70	0.70
7. To try to get a reaction from someone, even if it's negative		0.79		0.64	0.66	0.58
8. To receive more attention from your parents or friends		0.85		0.73	0.84	0.89
15. To let others know how desperate you were		0.74		0.53	0.57	0.55
17. To get your parents to understand or notice you		0.65		0.69	0.84	0.84
20. To get help		0.59		0.50	0.53	0.45
1. To avoid school, work, or other activities			0.75	0.49	0.61	0.58
5. To avoid doing something unpleasant you don't want to do			0.84	0.58	0.77	0.87
9. To avoid being with people			0.59	0.40	0.43	0.33
13. To avoid punishment or paying the consequences			0.70	0.41	0.48	0.32

*Factor 1, emotion regulation, Factor 2, attention seeking, Factor 3, social avoidance. ID, Item-rest correlation*.

#### Confirmatory Factor Analysis

CFA was used to test the goodness of fit of competing models of the structure of the FASM function (the 18-item, the 17-item, the 16-item, and the final 15-item) in sample 2. As shown in Table 4, the 15-item three-factor CFA model showed a good model fit (χ^2^ = 183.62, df = 87, *p* < 0.001, TLI = 0.90, CFI = 0.93, GFI = 0.92, AGFI = 0.90, SRMR = 0.06, RMSEA = 0.06, AIC = 12586.32) and all items presented high factorial weights (>0.42, Table 3). Additionally, ESEM was performed on the three-factor model. The three-factor ESEM model also showed a good model fit (χ^2^ = 86.55, df = 63, *p* = 0.026, TLI = 0.96, CFI = 0.98, GFI = 0.97, AGFI = 0.95, SRMR = 0.03, RMSEA = 0.03, AIC = 12556.25), and all items presented factor loadings > 0.32 (Tables 3, 4).

**Table 4:** FASM function confirmatory factor analysis model comparisons (*N* = 302).

**Model**	**χ^2^ value**	**χ^2^ *df***	**χ^2^ *p-value***	**CFI**	**GFI**	**AGFI**	**SRMR**	**RMSEA**	**AIC**
**CFA**
18-item	377.71	132	<0.001	0.84	0.83	0.79	0.08	0.08	15138.76
17-item(“item 18” removed)	317.73	116	<0.001	0.87	0.86	0.82	0.07	0.07	14263.79
16-item (“Item 6” removed)	271.12	101	<0.001	0.88	0.87	0.84	0.08	0.07	13501.52
15-item (“Item 11” removed)	183.62	87	<0.001	0.93	0.92	0.90	0.06	0.06	12586.32
**ESEM**
15-item	86.55	63	0.026	0.98	0.97	0.95	0.03	0.03	12556.25

**Figure 2:**
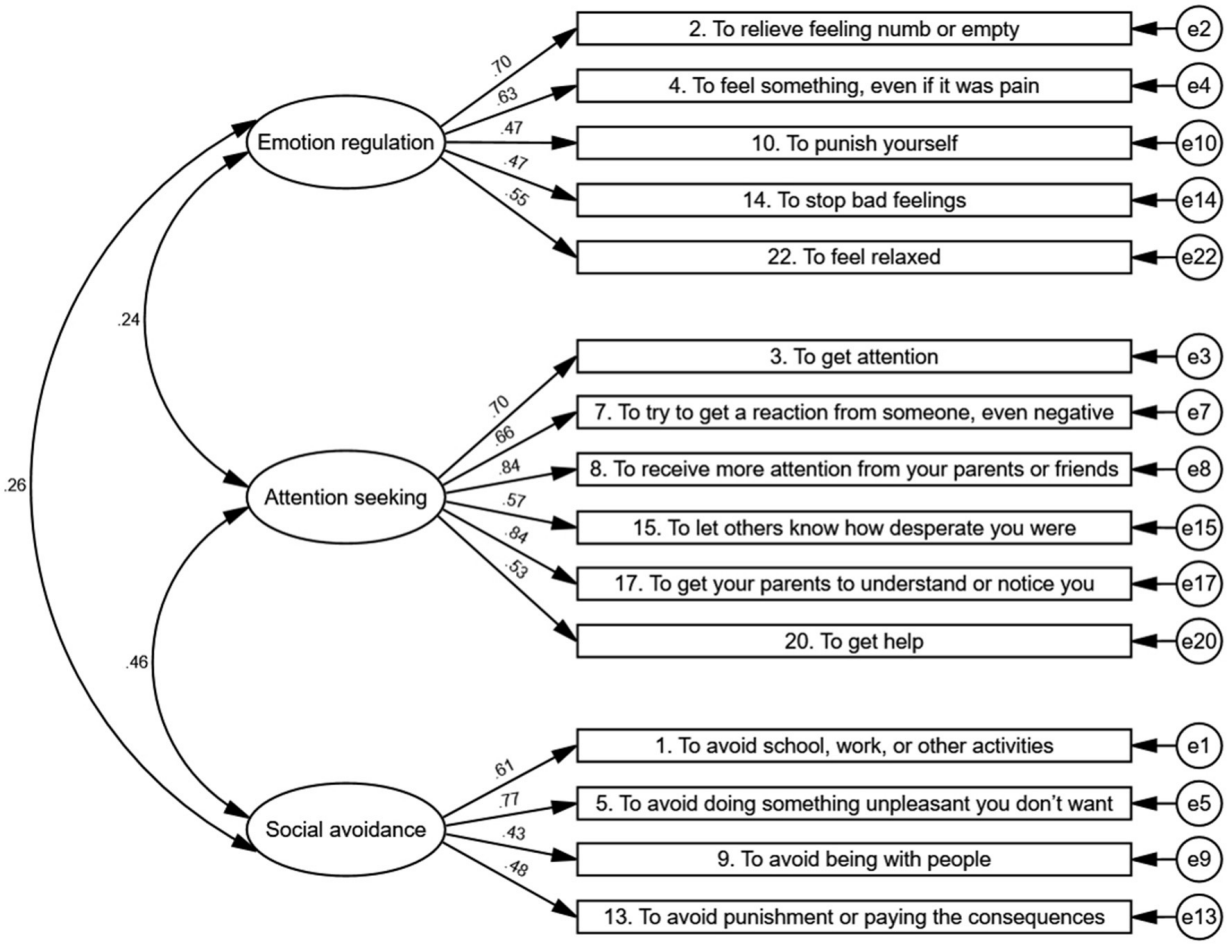
Confirmatory factor analysis.

On the basis of the results of the EFA, CFA, and ESEM, the C-FASM function checklist contained 15 items, and the indices suggested that a three-factor model could fit the function of C-FASM. The first factor mainly addressed emotion regulation function, which meant individuals engaging in NSSI to decrease or increase their effect, including the following items: items 2, 4, 10, 14, and 22. The second factor referred to attention seeking. It included explanations for individuals engaging in NSSI to increase social support and receive help and attention from others, including items 3, 7, 8, 15, 17, and item 20. The third factor addressed social avoidance, which referred to avoiding social demands, including items 1, 5, 9, and 13 (Figure 2).

Above all, the C-FASM including 10 items of the method checklist (including items 1, 2, 3, 4, 5, 6, 7, 8, 9, 10, 11, 13, 14, 15, 17, 18, 20, and 22; shown in Table 1), 15-item function checklist (including items 1, 2, 3, 4, 5, 7, 8, 9, 10, 13, 14, 15, 17, 20, and 22; shown in Table 2), and other characteristics of NSSI (including the age of onset of self-injury, amount of time they thought before engaging in self-injury, the degree of physical pain, and whether or not use of alcohol or drugs during self-injury). The Chinese translation of C-FASM was shown in Supplementary Table 2.

### Reliability of C-FASM

Two weeks after the first questionnaire was completed, a retest of the FASM was conducted on the adolescents who agree to complete the questionnaire a second time (*N* = 65).

The internal consistency (Cronbach's α and McDonald's Ω) indices and test–retest reliability for the C-FASM were shown in Table 5. The internal consistency indices revealed good internal consistency for the method checklist (α = 0.81, Ω = 0.80) and function checklist (α = 0.80, Ω = 0.76). The retest reliability of the method checklist was 0.79 and that of the function checklist was 0.87. The retest reliabilities of each factor were as follows: emotion regulation = 0.80, attention seeking = 0.83, and social avoidance = 0.61. The results indicated that the C-FASM is a reliable instrument to assess the method, frequency, and function of NSSI behaviors of Chinese adolescents over the past 12 months.

**Table 5:** Internal consistency and test-retest reliability of the C-FASM.

	**Internal consistency**		**Test-retest**
			**reliability**
	**Cronbach's α**	**McDonald's omega**	
Method checklist	0.81	0.80	0.79
Function checklist	0.80	0.76	0.87
Emotion regulation	0.71	0.71	0.80
Attention seeking	0.84	0.85	0.83
Social avoidance	0.70	0.70	0.61

### Criterion Validity of C-FASM

Criterion validity of the instrument was assessed by calculating the correlations between the C-FASM scores and the DSHI score (Table 6). C-FASM method checklist was significantly positively correlated with the DSHI (*r* = 0.84, *p* < 0.001), indicating that the C-FASM had good concurrent validity.

**Table 6:** Correlations among C-FASM and DSHI.

	**1**	**2**	**3**	**4**	**5**	**6**
1. FASM-method frequency	-					
2. FASM-function score	0.34^**^	-				
3. Emotion regulation score	0.44^**^	0.66^**^	-			
4. Attention seeking score	0.13^*^	0.78^**^	0.17^**^	-		
5. Social avoidance score	0.16^**^	0.69^**^	0.20^**^	0.42^**^	-	
6. DSHI	0.84***	0.34^**^	0.40^**^	0.15^**^	0.18^**^	-

* *p < 0.01*,

** *p < 0.001. DSHI, score of Deliberate Self-Harm Behavior Inventory*.

## Discussion

To our knowledge, the current study was the first study to develop and investigate the psychometric properties of the C-FASM for Chinese adolescents. In addition, this study examined the structure of C-FASM functions in a clinical adolescent sample. Two findings were found in the study. First, our findings suggested that the C-FASM was composed of a 10-item method checklist, a 15-item function checklist of NSSI behaviors, and other characteristics of NSSI, with acceptable to good model fits. Second, converging evidence from three different factor analyses indicated that the functions of NSSI were well captured by a three-factor structure with satisfying factor loadings, namely, emotion regulation, attention seeking, and social avoidance.

### Characteristics of NSSI Methods Among Chinese Adolescents

Our findings showed that the method of “cut or carved on your skin” was the most common method for adolescents who engaged in NSSI, which was in line with the previous studies investigated in Hongkong (33) and western countries (3). However, “Burned your skin” and “Erased your skin” were not common among Chinese adolescents with NSSI. An interesting finding was the high prevalence of the method of “Punched walls or objects,” with a report rate of 69.2% in this sample. That indicated the difference in self-injury methods between adolescents under different cultural backgrounds.

### Factorial Structure of the C-FASM NSSI Function

One of the main goals of this study was to investigate the factorial structure of the NSSI function through EFA, CFA, and ESEM. The functional model of FASM provided insight into possible reasons for engaging in NSSI (9). Over the two decades, researchers have proposed a series of theories to explain why people engage in NSSI. The two-factor model and four-factor model of the FASM function developed by Nock and Prinstein were the most plausible theoretical models to address the reasons for engaging in NSSI. According to the two-factor model, the causes of NSSI included automatic reinforcement (i.e., self-reinforcement; e.g., emotional regulation) and social reinforcement (i.e., reinforced by others; e.g., attention and avoidance). The four-factor functional model derived from the two-factor model, including automatic positive reinforcement, automatic negative reinforcement, social positive reinforcement, and social negative reinforcement. Interestingly, our findings showed a three-factor model with a good model fit. The three-factor model named emotion regulation, attention seeking, and social avoidance, which was close to previous findings among Hongkong high school students (18). The model gave rise to a potential reliable structure of NSSI function that existed across both clinical and non-clinical populations in the Chinese cultural context. Future studies are needed to investigate this three-factor structure in other cultural backgrounds.

Emotion regulation factor refers to adolescent's aim of NSSI to alleviate acute negative emotion or aversion arousal and increase their positive feelings. This factor was basically in accord with the automatic reinforcement of the two-factor model proposed by Nock and Prinstein (34) and intrapersonal functions of the two-factor model proposed by Klonsky and Glenn (6). Although most adolescents in our sample endorsed different functions, emotion regulation was the most common reason endorsed by adolescents, which was consistently suggested in many previous studies (9). In our sample, over 70% of the adolescents reported that they had engaged in NSSI to regulate their emotion, and the most endorsed reason was “To stop bad feelings” (87.9%). The results suggested that adolescents with NSSI may generally perform poorly in emotional management or regulation (35) and therefore lacked effective strategies to cope with emotional distress (36). Previous studies mentioned that NSSI was more likely to be seen as a solution to reduce distress (10). The emotion regulation factor endorsed by most adolescents in this study might confirm this theory to some extent. It indicated that emotional tolerance and regulation improvement might benefit when emotional regulation is an individual's NSSI function.

Besides the emotion regulation factor, attention-seeking and social avoidance factors were essential functions endorsed by Chinese adolescents. The attention-seeking factor meant that adolescents engaged in NSSI to seek social support, receive attention, or get help from others. It was closely related to positive social reinforcement, social impact, and social influence, as found in previous research (8, 18, 34, 37). However, the social avoidance factor referred to avoiding social demands, closely related to the social negative reinforcement factor of the four-factor model, highlighting that individuals with NSSI would cope with adversities through avoidant strategies (38).

#### The Gender Disbalance in Our Sample

A high female:male ratio of about 5:1 was found in our study. According to a meta-analysis, NSSI was more commonly seen among females than males (39). Tang et al. (5) have reported a higher prevalence of NSSI among girls than boys in China. Some earlier researches conceptualized NSSI as a “female” problem due to the large discrepancy between females and males among psychiatric patients (40). Our findings were in line with those studies. Moreover, our study showed a slightly different in NSSI methods and frequency between females and males. The details could be found in the previous study from our research group (41), which was consistent with some previous studies. For example, McManus et al. (42) reported that females who engaged in NSSI had roughly twice the odds of getting medical or psychological service than males had. Van et al. (43) observed the differences in NSSI methods in males and females.

### Limitation

To the best of our knowledge, this study is the first study to develop the C-FASM and test the validity and reliability of the C-FASM. However, the study has several limitations. First, it should be acknowledged that all the measures in this study were self-reported, which might be subjective. Second, the sample of this study only includes the patients coming from the hospital's psychiatric department, which may limit the use in the general population. Third, individuals with suicidal ideation may employ different functions than individuals without suicidal ideation, which should be further explored.

## In Conclusion

The findings indicated that the C-FASM presented adequate validity and reliability. The C-FASM can be helpful to both research and clinical measurement, contributing to subsequent investigations focused on adolescents and understanding the complex phenomenon of NSSI. Training young people to cope with stress and establish an attitude of actively seeking help may increase their confidence in dealing with difficulties, thereby reducing the occurrence of NSSI. At the same time, our findings yield great significance in guiding clinical intervention. Clinicians can formulate targeted interventions based on the three functions of NSSI.

## Data Availability Statement

This data can be found here: The raw data supporting the conclusions of this article will be made available by the authors, without undue reservation.

## Ethics Statement

The studies involving human participants were reviewed and approved by the Institutional Review Board (IRB) of the Shenzhen Kangning Hospital. Written informed consent to participate in this study was provided by the participants' legal guardian/next of kin.

## Author Contributions

YZ and DQ designed the study. LM, FZ, TZ, KH, YZ, and CL collected the data. YW and DQ wrote the manuscript. YW and HB analyzed the data. ZZ reviewed and edited the manuscript. YZ supervised all aspects of collection, analysis, and interpretation of the data. All authors contributed to and have approved the final manuscript.

## Funding

This work was supported by the Sanming Project of Medicine in Shenzhen (No. SZSM202011014), Shenzhen Fund for Guangdong Provincial High-level Clinical Key Specialties (No. SZGSP013), Shenzhen Key Medical Discipline Construction Fund (no.SZXK072), and Shenzhen Science and Technology Research and Development Fund for Sustainable development project (No. KCXFZ20201221173613036). The funders had no role in the study design, data collection, analysis and interpretation, writing of the report, and the decision to submit the article for publication.

## Conflict of Interest

The authors declare that the research was conducted in the absence of any commercial or financial relationships that could be construed as a potential conflict of interest.

## Publisher's Note

All claims expressed in this article are solely those of the authors and do not necessarily represent those of their affiliated organizations, or those of the publisher, the editors and the reviewers. Any product that may be evaluated in this article, or claim that may be made by its manufacturer, is not guaranteed or endorsed by the publisher.
