# Supplementary material for: Psychometric Properties of the Chinese Version of the Functional Assessment of Self-Mutilation (FASM) in Chinese Clinical Adolescents
Source: Front Psychiatry. 2022 Jan 26;12:755857. doi: 10.3389/fpsyt.2021.755857 (PMC8826685; doi:10.3389/fpsyt.2021.755857)
Supplement: Supplementary file 1 [file Data_Sheet_1.docx]

Table S1. Demographic characteristics of adolescents in sample 1 and sample 2.

| Characteristics | Total(N=621) | Sample 1(N=319) | Sample 2(N=302) | F/χ2 | *P*-value |
| --- | --- | --- | --- | --- | --- |
| Age(yrs), Mean(SD) | 15.0±1.7 | 15.1±1.7 | 15.0±1.6 | 0.09 | 0.768 |
| Sex, N(%) |  |  |  |  |  |
| boy | 93(15.0) | 52(16.3） | 41(13.6) |  |  |
| girl | 528(85.0) | 267(83.7) | 261(86.4) | 0.90 | 0.342 |
| Residential area, N(%) |  |  |  |  |  |
| urban | 197(31.7) | 103(32.3) | 94(31.1) |  |  |
| rural | 424(68.3) | 216(67.7) | 208(68.9) | 0.09 | 0.756 |
| Annual household income(CNY), N(%) | |  |  |  |  |
| <80000 | 250(40.3) | 131(41.1) | 119(39.4) |  |  |
| 80,000~ | 266(42.8) | 135(42.3) | 131(43.4) |  |  |
| ≧200,000 | 105(16.9) | 53(16.6) | 52(17.2) | 0.41 | 0.938 |
| Only-child, N(%) | 184(29.6) | 103(32.3) | 81(26.8) | 2.22 | 0.136 |

Table S2 Chinese version of FASM

**自残功能评估量表**

**（Functional Assessment of Self-Mutilation，FASM)**

**A:自伤方式：**

在过去的一年中，你是否做过以下故意伤害自己的行为，如有请在对应条目选择“有”（√），并在频率选项填写（次数），在是否接受过治疗选项填写“是”或“否”。

假如没有该项自伤行为，请选择“无” （√），频率及是否接受过治疗选项不需填写。

***接受过治疗：**特指因为自伤而接受的比如缝针或需到医疗机构处理的治疗。

|  | **无** | **有** | **多少次？（频率）** | **是否接受过治疗?** |
| --- | --- | --- | --- | --- |
| 1. 故意割伤或划伤皮肤 |  |  |  |  |
| 1. 故意打自己 |  |  |  |  |
| 1. 故意拽自己的头发 |  |  |  |  |
| 1. 故意用尖锐物体在身上刺/刻字或图案等 |  |  |  |  |
| 1. 故意刺激伤口妨碍愈合 |  |  |  |  |
| 1. 故意把物体刺入皮肤或指甲 |  |  |  |  |
| 1. 故意咬伤自己（如嘴或嘴唇等） |  |  |  |  |
| 1. 故意抓伤自己以致出血 |  |  |  |  |
| 1. 故意刮伤自己的皮肤 |  |  |  |  |
| 1. 用拳头击打或用头撞硬物 |  |  |  |  |
| 1. 其他故意伤害自己的行为（请填写具体方式）： |  |  |  |  |

**B. 如果过去一年中没有上述行为，你是否曾经有过上述任何一种行为？**

□是

□否

**如果在过去一年有上述任何一种行为，请填写以下问题 (C-H)：**

**C: 当做上述任何行为时，你是想自杀吗？**

□是

□否

**D: 在真的实施上述行为之前，你考虑了多久？**

□没有考虑

□几分钟

□小于60分钟

□大于1小时但小于24小时

□大于1天但小于1周

□大于1周

**E: 你是否曾在吸毒或饮酒的时候实施上述行为？**

□是

□否

**F: 你在自伤的过程中是否感受到疼痛？**

□重度疼痛

□中度疼痛

□轻度疼痛

□没有疼痛

**G: 你第一次这样伤害自己是几岁？ _________**

**H: 你是否曾因为以下原因伤害自己？（勾选所有符合的条目）**

| **原 因** | **从不** | **偶尔** | **有时** | **经常** |
| --- | --- | --- | --- | --- |
| 1. 为了逃避上学、工作或其他活动 | **0** | **1** | **2** | **3** |
| 1. 为了缓解麻木感或空虚感 | **0** | **1** | **2** | **3** |
| 1. 为了引起注意 | **0** | **1** | **2** | **3** |
| 1. 为了有一些感觉，哪怕是疼痛 | **0** | **1** | **2** | **3** |
| 1. 为了逃避自己不想做的讨厌事 | **0** | **1** | **2** | **3** |
| 1. 为了获得别人的回应，哪怕是负面回应 | **0** | **1** | **2** | **3** |
| 1. 为了从父母或朋友那里获得更多的关注 | **0** | **1** | **2** | **3** |
| 1. 为了避免跟人待在一起 | **0** | **1** | **2** | **3** |
| 1. 为了惩罚自己 | **0** | **1** | **2** | **3** |
| 1. 为了逃避惩罚或承担后果 | **0** | **1** | **2** | **3** |
| 1. 为了停止不好的感受 | **0** | **1** | **2** | **3** |
| 1. 为了让他人知道自己有多绝望 | **0** | **1** | **2** | **3** |
| 1. 为了让父母理解或注意自己 | **0** | **1** | **2** | **3** |
| 1. 为了获得帮助 | **0** | **1** | **2** | **3** |
| 1. 为了放松 | **0** | **1** | **2** | **3** |
| 1. 其他原因？（填写具体原因）：______________________________ | **0** | **1** | **2** | **3** |
